# Morphological changes in starch grains after dehusking and grinding with stone tools

**DOI:** 10.1038/s41598-019-38758-6

**Published:** 2019-02-20

**Authors:** Zhikun Ma, Linda Perry, Quan Li, Xiaoyan Yang

**Affiliations:** 1 Key Laboratory of Cultural Heritage Research and Conservation, School of Cultural Heritage, Northwest University, Xi’an, 710069 China; 2 Laboratory of Land Surface Pattern and Simulation, Institute of Geographic Sciences and Natural Resources Research, Chinese Academy of Sciences, Beijing, 100101 China; 3 The Foundation for Archaeobotanical Research in Microfossils, Fairfax, VA 22038 USA; 4 Department of Anthropology, George Washington University, Washington, DC 20052 USA; 5 Key Laboratory of Alpine Ecology, CAS Center for Excellence in Tibetan Plateau Earth System Sciences, Institute of Tibetan Plateau Research, Chinese Academy of Sciences, Beijing, 100101 China

## Abstract

Research on the manufacture, use, and use-wear of grinding stones (including slabs and mullers) can provide a wealth of information on ancient subsistence strategy and plant food utilization. Ancient residues extracted from stone tools frequently exhibit damage from processing methods, and modern experiments can replicate these morphological changes so that they can be better understood. Here, experiments have been undertaken to dehusk and grind grass grain using stone artifacts. To replicate ancient activities in northern China, we used modern stone tools to dehusk and grind twelve cultivars of foxtail millet (*Setaria italica*), two cultivars of broomcorn millet (*Panicum miliaceum*) and three varieties of green bristlegrass (*Setaira viridis*). The residues from both used and unused facets of the stone tools were then extracted, and the starch grains studied for morphological features and changes from the native states. The results show that (1) Dehusking did not significantly change the size and morphology of millet starch grains; (2) After grinding, the size of millet starch grains increases up to 1.2 times larger than native grains, and a quarter of the ground millet starch grains bore surface damage and also exhibited distortion of the extinction cross. This indicator will be of significance in improving the application of starch grains to research in the functional inference of grinding stone tools, but we are unable to yet distinguish dehusked forms from native.

## Introduction

From the Late Paleolithic to the Neolithic period, the use of ground stone tools was commonplace throughout the world. In recent years, research including ethnoarchaeology^1,2^, archaeological typology^1^, simulation experiments^3,4^, use-wear analysis^3,5,6^, residue analysis^6–8^, and statistical analysis^4^ have led scholars to hypothesize that ground stone tools were used mainly for food processing and preparation. Commonly cited plant food uses include dehusking and grinding cereals, and other proposed uses including paint processing, production of medicines, and tanning^1–11^.

As a method that extracts residues from the surfaces of used tools, starch grain analysis provides direct evidence for ancient processing methods and plant use^6–8,12,13^. These residues, however, frequently exhibit damage from processing with the tools that are being studied. Experiments that replicate these ancient processes have shown that both the size and morphology of starch grains can change after dehusking and grinding with stone tools^6,14,15^. Modern reference collections, however, predominantly consist of native, undamaged starch grains^16^. Some research has been completed on the morphological changes of starches from wheat (*Triticum aestivum*), foxtail millet (*Setaria italica*), legumes and other crops after grinding^14,17^. Grinding causes damage to starch grains, and is observable in both the morphology (such as a fractured surface or irregular outline) and extinction cross^17^. However, we have found that, for the species of plants we encounter most frequently in our samples, the lack of precisely defined changes that are mathematically analyzed has hindered the identification of the damaged portions of our assemblages.

Of the archaeological sites thus far identified in northern China, 90 have yielded lithic grinding tools^18^. At the early Neolithic sites of Nanzhuangtou, Donghulin and Zhuannian and the mid-Neolithic sites of Cishan, Xinglonggou and Peiligang, all in northern China, archaeobotanical assemblages of both macro- and micro-remains recovered from cultural deposits and ground stone tools are dominated by millets^19–22^. Because the archaeobotanical assemblages from these sites are key in understanding the transition from cultivation to domestication of these plants in this region, our studies focused on this suite of plants. We used stone implements to dehusk and grind samples from foxtail millet (*Setaria italica*), broomcorn millet (*Panicum miliaceum*) and green bristlegrass (*Setaria viridis*), the three most commonly occurring species of millets at these sites. The starch grains in this simulation experiment were extracted via sonication, examined under the microscope, measured and then the statistical analysis was performed on the measured grains. Our statistical analyses of the sonication demonstrate that ancient starch residues from these plants can now be accurately identified in assemblages that include damaged grains.

## Results

### Starch grain analysis for seeds from modern millet seeds

The morphological features of starch grains extracted from the modern millet seeds are consistent with results published previously^23^.

#### Starch grain analysis for seeds of foxtail millet

**Figure 1:**
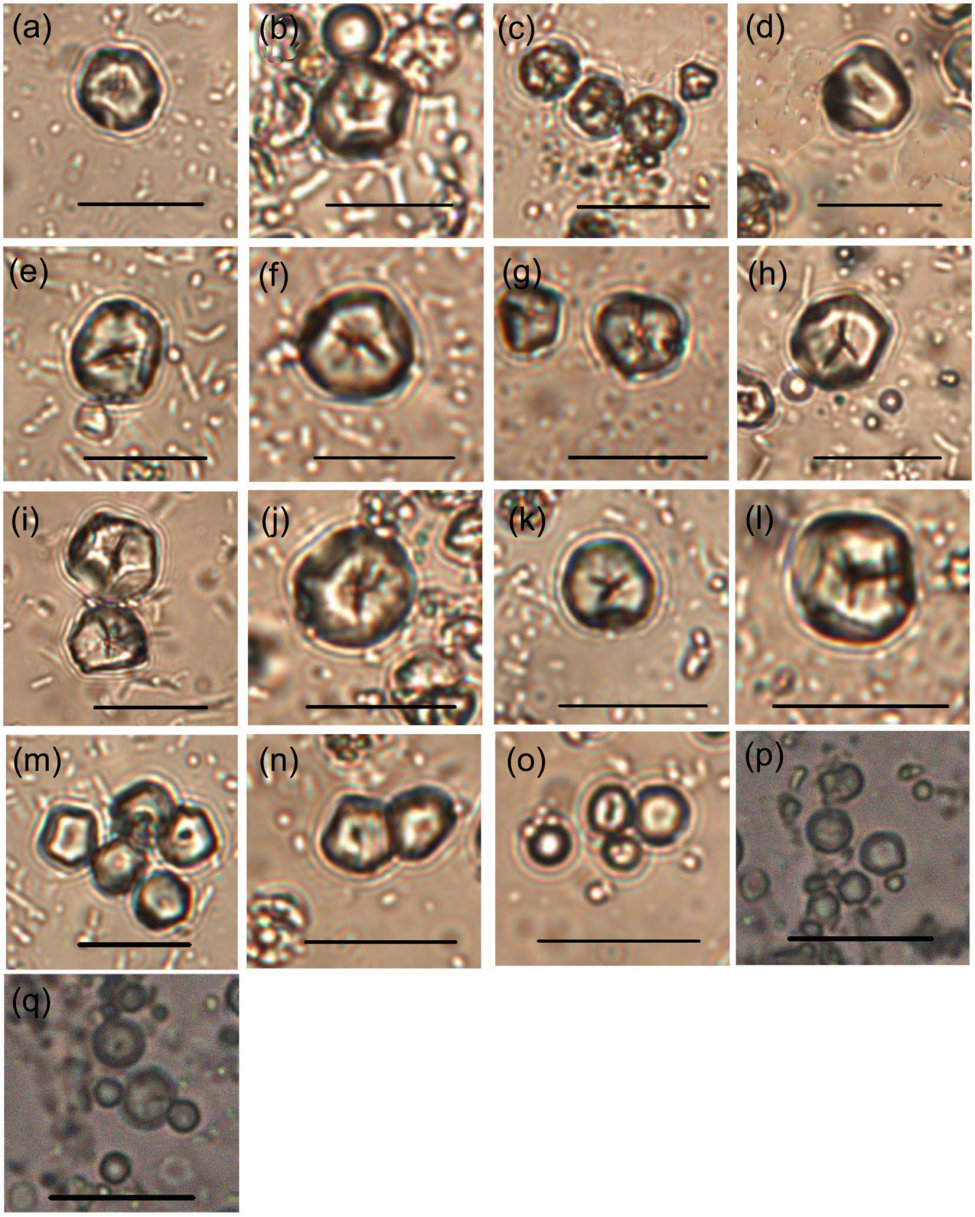
Native starch grains from foxtail millet, broomcorn millet and green bristlegrass. Scale bar: 20 μm. The cultivar name of seventeen millets. (**a**) Qimengkelake. (**b**) Gouzhuagu. (**c**) Maomaodouzhimaliang. (**d**) Kaoshanmen. (**e**) Zhushushu. (**f**) Mingu. (**g**) Yugu. (**h**) Qutangxiaomi. (**i**) Huangkegouweisu. (**j**) Zheng No. 468. (**k**) Shaonong No. 11. (**l**) Baigu. (**m**) Jishu No. 1. (**n**) Jinshu No. 4. (**o**) Jinsegouweicao. (**p**) Judagouweicao. (**q**) Zhouyegouweicao.

Surface morphology: The surface morphologies of all the recovered starches from the twelve different cultivars of foxtail millets are almost identical. The common features of the processed starches include a polyhedral shape, centric hilum, absence of lamellae, and smooth surfaces. Deeply crossed, or winged, astroid and Y-shaped fissures were noted in some grains. Radiating lines occur commonly from the center to the edge on the surfaces of processed grains (Fig. 1a–l).

**Figure 2:**
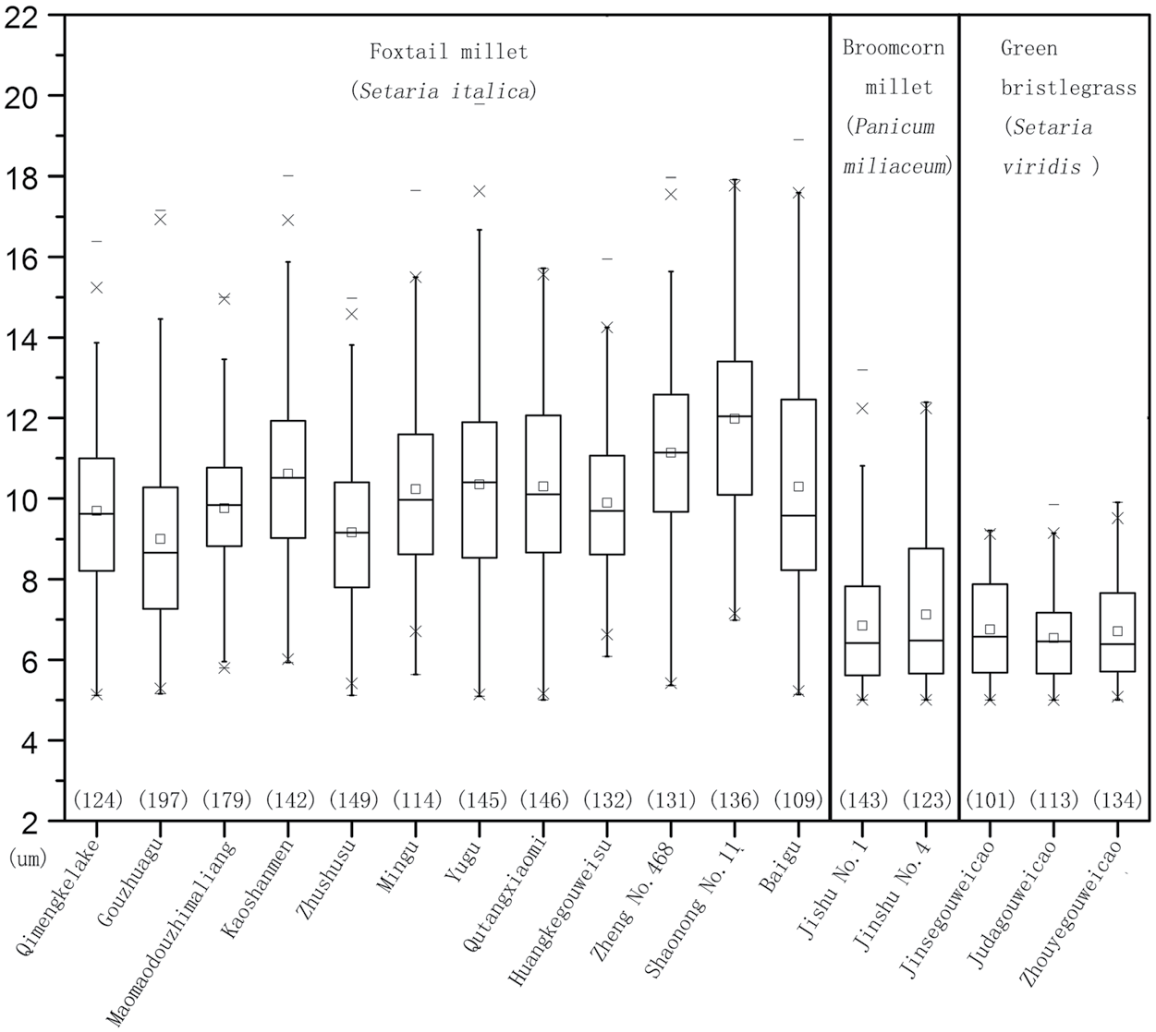
The number and size distribution of starch grains from foxtail millet, broomcorn millet and green bristlegrass.

Mean size: In total, 1704 starch grains from 12 foxtail millet samples were measured. The mean maximum length measurements fall between 9.0 μm and 12.2 μm (Mean, 9.0~12.2 μm; Range, 5.0–19.8 μm; Fig. 2; Table 1). The number of starches measuring >14.0 μm is 92, comprising 5.4% of the total (Fig. 2, Table 1).

**Table 1:** Measurements of modern millet starch grains.

Lab No.	Name	Latin name	Weight (gram)	Place of origin (province)	Sample source*	mean size (um)	minimum size (um)	maximum size (um)	size >14 um (*n*)	Total (*n*)
A	Qimengkelake	*Setaria italica*	9.5	Xinjiang	CAAS	9.7 ± 2.0	5.1	16.4	2	124
B	Gouzhuagu	*Setaria italica*	11.7	Hebei	CAAS	9.0 ± 2.4	5.1	17.2	7	197
C	Maomaodouzhimaliang	*Setaria italica*	5.9	Shanxi	CAAS	9.8 ± 1.7	5.8	15.0	2	179
D	Kaoshanmen	*Setaria italica*	21.5	Jiangsu	CAAS	10.6 ± 2.4	5.9	18.0	12	142
E	Zhushusu	*Setaria italica*	10.2	Heilongjiang	CAAS	9.2 ± 2.0	5.1	15.0	2	149
F	Mingu	*Setaria italica*	16.6	Fujian	CAAS	10.2 ± 2.2	5.6	17.7	7	114
G	Yugu	*Setaria italica*	24.6	Henan	CAAS	10.3 ± 2.6	5.3	19.8	11	145
H	Qutangxiaomi	*Setaria italica*	26.6	Guangxi	CAAS	10.3 ± 2.2	5.0	15.7	4	146
I	Huangkegouweisu	*Setaria italica*	21.7	Hainan	CAAS	9.9 ± 1.8	6.1	16.0	3	132
J	Zheng No. 468	*Setaria italica*	17.3	Henan	CAAS	11.1 ± 2.4	5.4	18.0	15	131
K	Shaonong No. 11	*Setaria italica*	12.0	Inner Mongolia	CAAS	12.2 ± 2.4	7.0	18.0	11	136
L	Baigu	*Setaria italica*	12.8	Tibet	CAAS	10.3 ± 3.1	5.1	18.9	16	109
M	Jishu No. 1	*Panicum miliaceum*	13.4	Hebei	Near Cishan site	6.8 ± 1.9	5.3	13.1	0	143
N	Jinshu No. 4	*Panicum miliaceum*	10.6	Shanxi	CAAS	7.1 ± 1.6	5.1	12.4	0	123
O	Jinsegouweicao	*Setaria glauca*	4.6	Beijing	Near CAAS	6.8 ± 1.1	5.1	9.2	0	101
P	Judagouweicao	*Setaria viridis*	9.7	Hebei	Near Cishan site	6.5 ± 0.9	5.2	9.8	0	113
Q	Zhouyegouweicao	*Setaria plicata*	10.3	Zhejiang	Near Shangshan site	6.7 ± 1.2	5.4	9.9	0	134

*Annotation: “CAAS” indicates a sample provided by the Chinese Academy of Agricultural Sciences.

#### Starch grain analysis for seeds of broomcorn millet

Surface morphology: The surface morphologies of the starch grains from the two broomcorn millet samples are almost identical. Polyhedral starch grains dominate the sample. The hila of the starch grains are centric, and 72% of the grains are unfissured. The remaining 28% grains have slight fissures through the hila, and a very few starch grains have transverse or Y-shaped fissures (Fig. 1m,n).

Mean size: More than 100 starch grains were measured for each sample for a total of 266 starches (mean, 6.9 ± 1.8 μm; range, 5.1–12.4 μm; Fig. 2).

#### Starch grain analysis for seeds of green bristlegrass

Surface morphology: All starch grains from the three samples of green bristlegrass have polyhedral or spherical shapes, centric hila, wrinkled surfaces and coarse edges. Few starches from green bristlegrass have fissures through the hila which vary in form (Fig. 1o–q).

Mean size: More than 100 starch grains were measured for each sample for a total of 348 starches (mean, 6.7 ± 1.1 μm; range, 5.1–9.9 μm; Fig. 2).

### Starch grains from residues on the surfaces of stones used for dehusking

#### Starch grains from dehusking foxtail millet

The starch grains extracted from residues on the surfaces of stones used to dehusk the seeds of foxtail millet are morphologically indistinguishable from native foxtail millet seed starches.

**Table 2:** Starch measurements from experimental stones*.

Lab No	Tool Type	Plant name	Length (cm)	Width (cm)	Lithology	Sampling spot	Data of starch grains recovered from stones after dehusking						Data of starch grains recovered from stones after grinding					
							mean size (um)	minimum size (um)	maximum size (um)	size >14um (*n*)	process time (minute)	Total (*n*)	mean size (um)	minimum size (um)	maximum size (um)	size >14um (*n*)	process time (minute)	Total (*n*)
a	slab	Qimengkelake	9.3	7.2	sandstone	Xindian village	11.4 ± 3.8	7.2	15.6	1	40	2	11.7 ± 2.8	6.3	27.6	31	10	149
	muller	Qimengkelake	6.2	3.9	sandstone	Xindian village	—	—	—	—		—	12.5 ± 3.1	7.1	25.3	25		150
b	slab	Gouzhuagu	7.5	6.7	sandstone	Xindian village	—	—	—	—	53	—	11.8 ± 3.3	6.1	26.1	21	21	150
	muller	Gouzhuagu	5.2	3.8	sandstone	Xindian village	14.5 ± 0.0	14.5	14.5	1		1	11.3 ± 2.7	6.0	27.9	32		161
c	slab	Maomaodouzhimaliang	10.2	8.7	sandstone	Xindian village	10.2 ± 2.8	6.1	15.3	1	38	3	12.2 ± 3.3	9.8	25.3	29	17	166
	muller	Maomaodouzhimaliang	6.2	5.7	shale	Xindian village	—	—	—	—		—	11.9 ± 2.8	6.1	24.9	36		186
d	slab	Kaoshanmen	5.5	4.3	shale	Xindian village	11.3 ± 1.0	10.3	12.3	0	76	2	12.6 ± 3.5	6.1	34.4	27	23	144
	muller	Kaoshanmen	3.7	2.6	sandstone	Xindian village	13.7 ± 0.0	13.7	13.7	0		1	13.1 ± 4.2	6.9	29.1	32		175
e	slab	Zhushusu	7.2	6.8	sandstone	Xindian village	10.8 ± 2.7	6.8	16.7	1	49	4	11.9 ± 3.0	6.8	26.7	33	11	167
	muller	Zhushusu	4.2	3.7	sandstone	Xindian village	8.3 ± 2.4	5.9	10.9	0		3	11.4 ± 2.4	6.0	30.9	31		159
f	slab	Mingu	8.4	6.8	sandstone	Xindian village	12.6 ± 2.5	10.5	14.2	0	68	3	11.8 ± 2.8	6.1	22.3	26	19	179
	muller	Mingu	5.8	3.2	sandstone	Xindian village	—	—	—	—		—	10.8 ± 3.0	7.1	21.9	21		179
g	slab	Yugu	8.6	7.4	sandstone	Xindian village	11.5 ± 0.0	11.5	11.5	0	83	1	13.6 ± 3.7	7.6	24.9	44	25	147
	muller	Yugu	6.1	5.6	limestone	Xindian village	12.1 ± 1.6	6.8	16.9	1		3	12.4 ± 2.9	8.1	23.6	56		178
h	slab	Qutangxiaomi	6.6	5.3	sandstone	Xindian village	11.6 ± 0.0	11.6	11.6	0	90	1	12.0 ± 2.8	6.9	21.2	37	27	167
	muller	Qutangxiaomi	5.1	4.6	sandstone	Xindian village	5.1 ± 0.0	5.1	5.1	0		1	11.8 ± 3.0	6.4	20.9	40		172
i	slab	Huangkegouweisu	6.2	5.7	sandstone	Xindian village	—	—	—	—	78	—	11.7 ± 2.7	6.8	22.7	18	23	144
	muller	Huangkegouweisu	3.7	3.1	sandstone	Xindian village	—	—	—	—		—	11.0 ± 2.7	5.9	21.6	23		152
j	slab	Zheng No. 468	8.3	4.2	sandstone	Xindian village	8.3 ± 1.3	5.2	9.9	0	40	3	12.0 ± 3.0	5.5	23.0	27	17	171
	muller	Zheng No. 468	4.9	3.7	sandstone	Xindian village	6.9 ± 0.0	6.9	6.9	0		1	11.6 ± 2.8	5.8	27.4	24		186
k	slab	Shaonong No. 11	6.3	5.8	sandstone	Xindian village	—	—	—	—	45	—	14.7 ± 3.4	7.9	29.6	27	15	156
	muller	Shaonong No. 11	3.8	2.9	sandstone	Xindian village	12.4 ± 2.4	10.7	14.0	1		2	15.4 ± 3.4	8.7	30.2	31		167
l	slab	Baigu	7.2	7.1	sandstone	Xindian village	7.2 ± 0.0	7.2	7.2	0	43	1	13.2 ± 4.0	6.9	29.4	37	14	172
	muller	Baigu	6.3	4.9	sandstone	Xindian village	—	—	—	—		—	12.9 ± 3.9	5.9	28.5	29		175
m	slab	Jishu No. 1	5.7	5.3	sandstone	Xindian village	7.8 ± 0.0	7.8	7.8	0	38	1	9.6 ± 2.7	5.1	15.5	9	16	168
	muller	Jishu No. 1	3.6	2.7	sandstone	Xindian village	—	—	—	—		—	9.3 ± 1.8	5.3	16.0	11		175
n	slab	Jinshu No. 4	6.7	5.3	sandstone	Xindian village	7.3 ± 1.8	5.5	9.1	0	27	2	9.1 ± 2.3	5.8	14.9	7	11	147
	muller	Jinshu No. 4	4.2	3.0	sandstone	Xindian village	—	—	—	—		—	8.5 ± 2.1	6.0	15.8	11		145
o	slab	Jinsegouweicao	7.6	6.3	sandstone	Xindian village	—	—	—	—	21	—	8.1 ± 2.0	5.5	12.3	0	8	146
	muller	Jinsegouweicao	3.7	2.8	shale	Xindian village	—	—	—	—		—	8.2 ± 2.1	5.7	11.8	0		145
p	slab	Judagouweicao	6.7	3.7	sandstone	Xindian village	—	—	—	—	40	—	7.9 ± 1.8	5.3	10.8	0	15	150
	muller	Judagouweicao	3.8	2.1	sandstone	Xindian village	—	—	—	—		—	7.7 ± 1.6	5.1	11.2	0		172
q	slab	Zhouyegouweicao	6.9	4.7	sandstone	Xindian village	—	—	—	—	52	—	8.0 ± 2.2	5.3	11.0	0	19	167
	muller	Zhouyegouweicao	4.5	2.8	sandstone	Xindian village	—	—	—	—		—	7.5 ± 1.7	5.1	12.0	0		168

*Annotation: No starch was collected from the unused facet of the sandstone tools from the group a∼q. “—” Indicates no starch was recovered from the sampled material.

**Figure 3:**
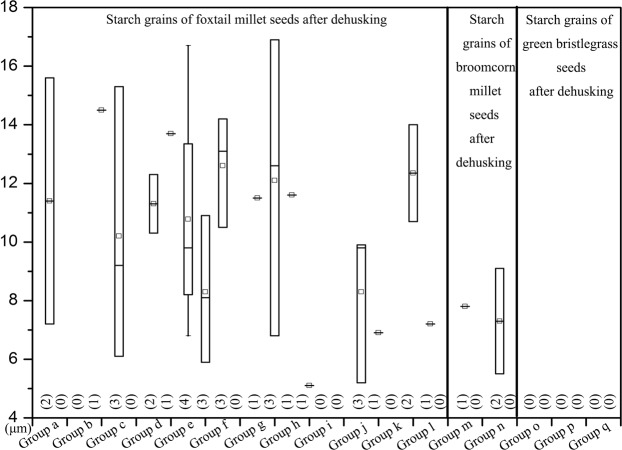
The number and size distribution of starch grains recovered from dehusking foxtail millet, broomcorn millet and green bristlegrass.

Mean size: All starch grains were measured for each sample. 32 starches from 12 samples were measured (mean, 5.1–14.5 μm; range, 5.1–16.9 μm; Table 2; Fig. 3).

#### Starch grains from dehusking broomcorn millet

The starch grains extracted from the residues on the surfaces of the stones used to dehusk broomcorn millet, are morphologically indistinguishable from native broomcorn millet seed starches.

Mean size: All starch grains were measured for each sample. 3 starches from 2 samples were measured (mean, 7.5 ± 1.8 μm; range, 5.5–9.1 μm; Table 2; Fig. 3).

#### Starch grains from dehusking green bristlegrass

No starch was recovered from the surface of the stone tools used to dehusk green bristlegrass (Table 2; Fig. 3).

### Starch grains from residues on the surface of stones used for grinding

#### Starch grains from grinding foxtail millet

Surface morphology: The starch grains extracted from residues on the surfaces of each set of stones used to grind foxtail millet can be divided into two types: A1 with an intact surface and A2 with a damaged surface. The starches of type A1 are morphologically identical to native foxtail millet seed starches. Type A2 starches have both surface damage and internal disruption that result in the disappearance or alteration of the hilum and fissure and a weakened extinction cross.

**Figure 4:**
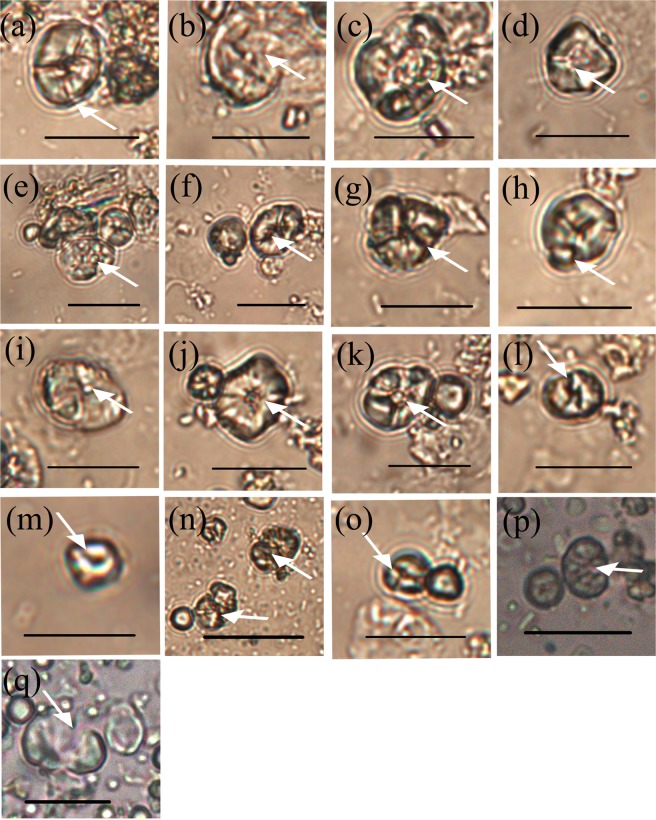
Damaged starch grains from foxtail millet, broomcorn millet and green bristlegrass after grinding. White arrows indicate the damaged areas. Scale bar: 20 μm. (**a**) Qimengkelake, the occurrence of some fissures radiating from the hilum. (**b**) Gouzhuagu, fractured surface. (**c**) Maomaodouzhimaliang, striations in different directions. (**d**) Kaoshanmen, irregular outline. (**e**) Zhushushu, rough surface. (**f**) Mingu, the occurrence of some fissures radiating from the hilum. (**g**) Yugu, irregular outline. (**h**) Qutangxiaomi, irregular outline. (**i**) Huangkegouweisu, broken surface. (**j**) Zheng No. 468, the occurrence of some fissures radiating from the hilum. (**k**) Shaonong No. 11, striations in different directions. (**l**) Baigu, extinction cross. (**m**) Jishu No. 1, extinction cross. (**n**) Jinshu No. 4, rough surface. (**o**) Jinsegouweicao, rough surface. (**p**) Judagouweicao, rough surface. (**q**) Zhouyegouweicao, fractured surface.

**Figure 5:**
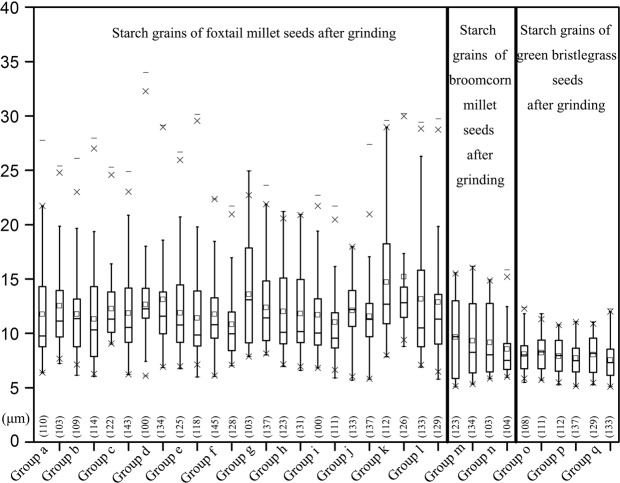
The number and size distribution of starch grains with an intact surface recovered from the grinding experiments with foxtail millet, broomcorn millet and green bristlegrass.

Mean size: In total, 3952 starch grains from 12 samples were measured, and, notably, type A2 starch grains with obvious damage and weakened extinction crosses (Fig. 4a–l) make up 27.0% (*n* = 1026) of the population. The remaining 73.0% of the starch grains fall into category A1 (*n* = 2926) within which 737 starches measure >14.0 μm (mean, 10.8–15.4 μm; range, 5.5–34.4 μm; Table 2; Fig. 5).

#### Starch grains from grinding broomcorn millet

Surface morphology: The starch grains extracted from residues on the surfaces of each set of stones used to grind broomcorn millet can be categorized into two types: B1 is morphologically identical to native broomcorn millet seed starch. Only 63% of type B1 starches have no fissure. B2 starches have a damaged surface, altered morphology, and the hilum and fissure have disappeared or been altered in 100% of the grains (Fig. 4m,n).

Mean size: In total, 635 starch grains including 464 type B1 and 171 type B2 from 2 group samples were measured. The mean sizes of type B1 starches were 8.8 ± 2.2 μm within which 38 starches measured >14.0 μm (mean, 8.8 ± 2.2 μm; range, 5.8–15.8 μm; Table 2; Fig. 5).

#### Starch grains from grinding green bristlegrass

Surface morphology: The starch grains extracted from residues on the surfaces of each set of stone tools after grinding green bristlegrass can be categorized into two types: C1, which are morphologically identical to native green bristlegrass seed starches, and type C2 with various morphologies and a disappearing or ambiguous hilum and fissure (Fig. 4o–q).

Mean size: In total, 948 starch grains from 3 group samples were measured. The mean sizes of 730 type C1 starches were 8.1 ± 2.1 μm (range, 5.5–12.3 μm; Table 2; Fig. 5).

## Discussion

### Starch grain analysis of foxtail millet, broomcorn millet and green bristlegrass

At the genus level, both the sizes and the morphological features of the native starch grains from foxtail millet, broomcorn millet and green bristlegrass overlap somewhat. Thus, a basic statistical assemblage analysis of a population of starch grains was used to separate these millet plants from one another: the green bristlegrass starch grains which have wrinkled surfaces and coarse edges are the smallest, the broomcorn millet starch grains which have smooth surfaces and fewer fissures are a little larger, and the foxtail millet starch grains which have smooth surfaces and astroid and Y-shaped fissures are the largest (Fig. 2 and Table 1). Only foxtail millet has starch grains measuring between 14.0 μm and 20 μm. Within the cultivars and varieties that we sampled, both the sizes and the morphological features of the native starch grains within all three species differed very little.

Our results from the analyses on these particular cultivars and varieties of millets are consistent with previous work in China^17,23^. When compared with starch data collected on millets from the United States, India and Africa, the basic shapes of the millet starch grains are consistent, however, the sizes of millet starch grains in this experiment are large^24–26^. We believe that this size difference may be an artifact of our measuring methods. Many scientists measure all the millet starches in a given sample, while we only measure the starches >5 μm. Nonetheless, at this time, we cannot be certain of the cause. Further research is necessary to determine if there may be size differences that can be attributed to the internal and external environments of the millet plants grown in different regions.

### Starch grains from the surfaces of stones used to dehusk millets

We recovered no residues from the unused facets of the tools, a result that is likely due to the lack of contact between the stone and the millet seeds. In contrast, 35 starch grains were extracted from the used facets of the stones (Table 2).

The morphological features, maximum, minimum, and average length of these 35 starch grains were compared with native millet starches, they were difficult if not impossible to distinguish from one another (p > 0.05) (Tables 2 and 3, Fig. 3). Dehusking does not seem to be an activity that produces enough friction to damage starch grains of millets in a distinctive way.

**Table 3:** The results of One-sample T test for the starches on the surface of stones and from whole millet seeds (SPASS 20.0)^*^.

	Test Value = 0000					
	t	df	Sig. (2-tailed)	Mean Difference	95% Confidence Interval of the Difference	
					Lower	Upper
Dehusking FM	0.936	31	0.357	568.75000	−671.0748	1808.5748
Grinding FM	28.479	2925	0.000	2288.3134	2130.7655	2445.8613
Seed FM	2.470	1700	0.014	142.64993	29.3825	255.9174
Dehusking BM	−2.407	2	0.138	−2533.3333	−7061.9193	1995.2527
Grinding BM	−5.468	463	0.000	−812.31804	−1104.2555	−520.3806
Seed BM	−28.75	266	0.000	−3024.1679	−3231.2767	−2817.0592
Dehusking GB	—	—	—	—	—	—
Grinding GB	−34.902	730	0.000	−2113.4646	−2232.3475	−1994.5816
Seed GB	−52.154	349	0.000	−3331.3394	−3456.9687	−3205.7101

*Annotation: “FM”, “BM” and “GB” indicates the foxtail millet, broomcorn millet and green bristlegrass; “—” indicates no starch was recovered from the sampled material; The length unit of starches in Table 3 is nanometer.

### Starch grains from the surfaces of stones used to grind millets

No starch grains were recovered from the unused facets of the stones. In contrast, 5535 starch grains, a very large number, were recovered from the samples from the used facets of the experimental tools. This total breaks down into 2690 starch grains from the mullers and 2845 from the slabs. The large numbers of starch grains deposited on the surfaces of these grinding tools indicate that ancient activities would result in similarly thick residues.

**Figure 6:**
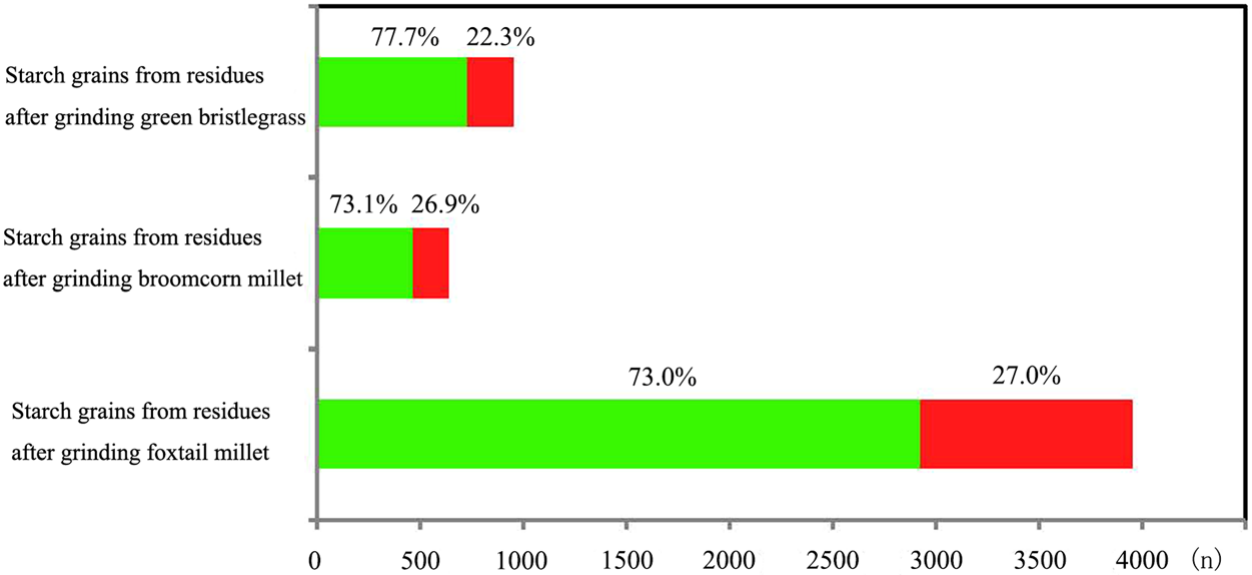
The number of identifiable and unidentifiable starch grains taken from grinding stones.

5535 starch grains were studied and measured for statistical analysis. Among all the samples and all the species, we found that the percentage of obvious, identifiable starch grains derived from foxtail millet, broomcorn millet and green bristlegrass were 73.0%, 73.1% and 77.7%; the percentage of unidentifiable starch grains with breakage and distorted morphological features were 27.0%, 26.9% and 22.3% (Fig. 6). Overall, 26% of millet starch grains bore damage from grinding that would be clearly recognizable in the archaeobotanical record (Fig. 6).

Green indicates the proportion of identifiable starch grains; Red indicates the proportion of unidentifiable starch grains.

Scholars typically analyze intact ancient starch grains to make identifications^6–8,12^. We believe, however, that damaged grains can also be taken into account when identifying the sources of larger assemblages. Previous studies have determined that domesticated foxtail millet is the only plant among the millets that produces starch grains measuring >14.0 μm^17,23^. However, starch grains measuring >14.0 μm also appear in both foxtail millet and broomcorn millet after grinding. Our analysis of 4120 intact millet starch grains taken from both the residues on the surface of stones and from whole millet seeds (Figs 2 and 5) demonstrates that, overall, the maximum length of millet starch grains after seed grinding can reach as much as 34.4 μm. This measurement is much larger than the maximum length of starch grains observed in intact, unprocessed, millet seeds (our maximum was 19.8 μm in Yugu). The mean size of millet starch grains after grinding tends to be 1.2 times greater than that of native grains, a statistically significant difference (P < 0.05) (Tables 2 and 3). Meanwhile, the percentage of starch grains measuring >14 μm increases from 5.4% to 18.6% in foxtail millet and 0% to 6.0% in broomcorn millet after grinding. At the genus level, our analysis of 4120 intact millet starch grains demonstrates that the green bristlegrass starch grains are small, the majority are <10 μm in length, and have wrinkled surfaces and rough edges; The size of broomcorn millet starch grains still lies solidly in the center of the data set; Only foxtail millet has starch grains larger than 16.8 μm after seed grinding (Fig. 5). That is to say, using an assemblage approach, we can distinguish wild millet (the majority are <10 μm in length, wrinkled surfaces and rough edges) and partial foxtail millet (>16.8 μm) from a mixed wild/domesticated millet assemblage after grinding.

## Conclusion

In this simulation experiment, ground stone tools were used to dehusk and grind twelve cultivars of foxtail millet, two cultivars of broomcorn millet and three varieties of green bristlegrass. We then extracted, counted, and studied the starch grains from residues collected from the surfaces of both the used and unused facets of the stones. Based upon the morphological and statistical mean diameters of the processed starch grains, initial criteria for their identification have been constructed. (1) After dehusking, the morphology of millets does not change in a manner that would be identifiable in the archaeobotanical record. (2) After grinding, the size of millet starches increases up to 1.2 times larger than native grains, and a quarter of the ground millet starch grains bore surface damage and also exhibited distortion of the extinction cross. (3) The number of starch grains deposited on the surfaces of stones was much higher after grinding than after dehusking. While taphonomic processes will certainly affect starch assemblages, the results of this study can be helpful in distinguishing tool function in assemblages derived from similar contexts.

Our data demonstrate that an assemblage approach, taking into account the proportions of damaged and large-sized starch grains, may be necessary in understanding the underlying composition of the millet population, and, thus, will assist in documenting the transition from wild to domesticated forms in the archaeobotanical record.

## Materials and Methods

### Materials

#### Mature ears from foxtail millet, broomcorn millet and green bristlegrass

**Figure 7:**
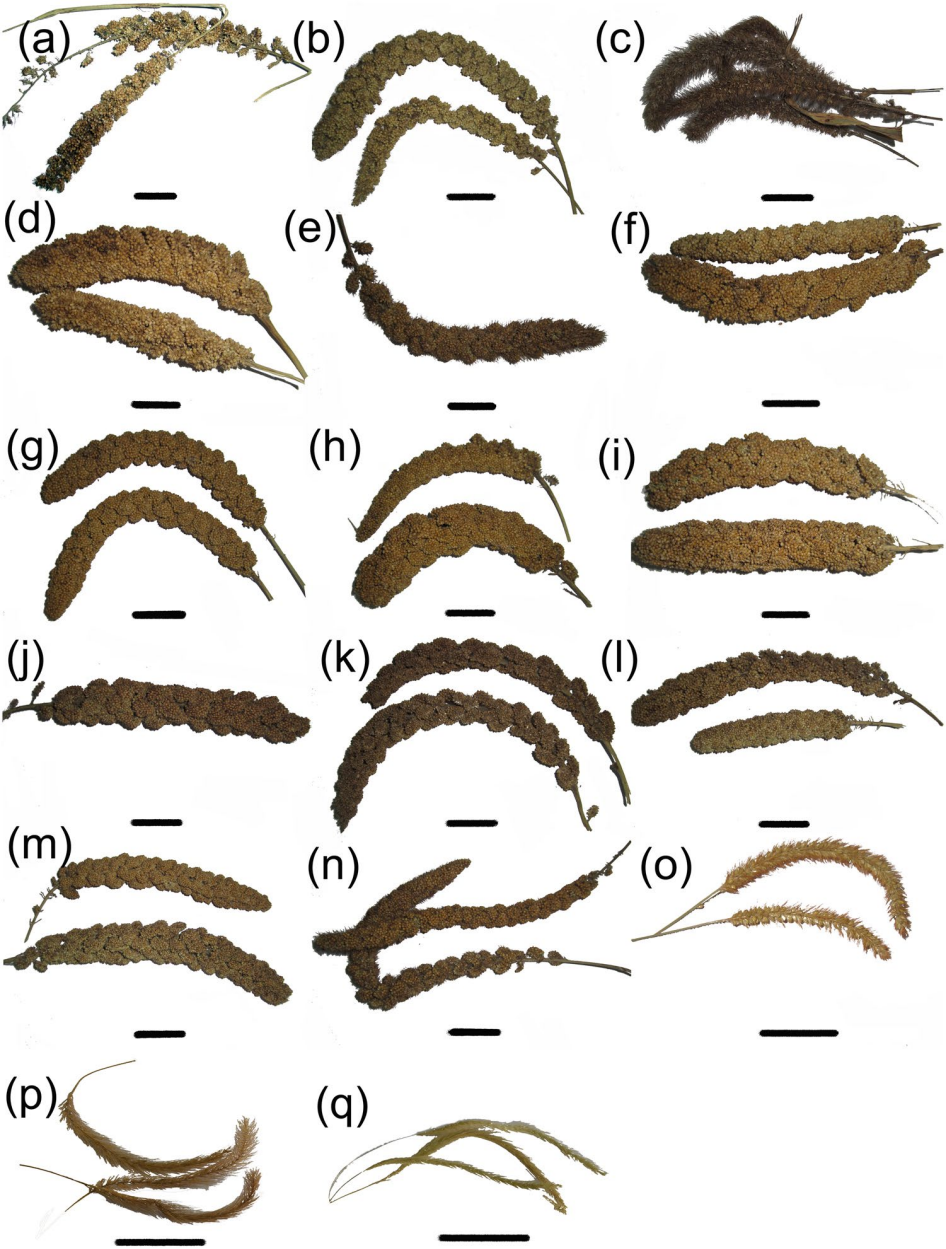
Ear samples from native foxtail millet, broomcorn millet and green bristlegrass. Scale bar: 2.5 cm. Cultivars and varieties. (**a**) Qimengkelake. (**b**) Gouzhuagu. (**c**) Maomaodouzhimaliang. (**d**) Kaoshanmen. (**e**) Zhushushu. (**f**) Mingu. (**g**) Yugu. (**h**) Qutangxiaomi. (**i**) Huangkegouweisu. (**j**) Zheng No. 468. (**k**) Shaonong No. 11. (**l**) Baigu. (**m**) Jishu No. 1. (**n**) Jinshu No. 4. (**o**) Jinsegouweicao. (**p**) Judagouweicao. (**q**) Zhouyegouweicao. All the images of foxtail millet, broomcorn millet and green bristlegrass were taken by Z. M.

Twelve cultivars of foxtail millet (Fig. 7a–l), one variety of green bristlegrass of genus *Setaria* (Fig. 7o), and one cultivar of broomcorn millet of genus *Panicum* (Fig. 7m) were collected in the farmland of Housangyuan Village, Changping District, Beijing (40°00′N, 116°00′E) in October 2011; One cultivar of broomcorn millet of genus *Panicum* (Fig. 7n) and one variety of green bristlegrass of genus *Setaria* (Fig. 7p) were collected in the field near the Cishan site, Hebei Province (36°34′N, 114°06′E) in September 2012; One variety of green bristlegrass of genus *Setaria* (Fig. 7q) was collected in the field near the Shangshan site, Zhejiang Province (29°27′N, 119°58′E) in September 2013 (Fig. 7 and Table 1**)**.

#### Modern grinding stones

**Figure 8:**
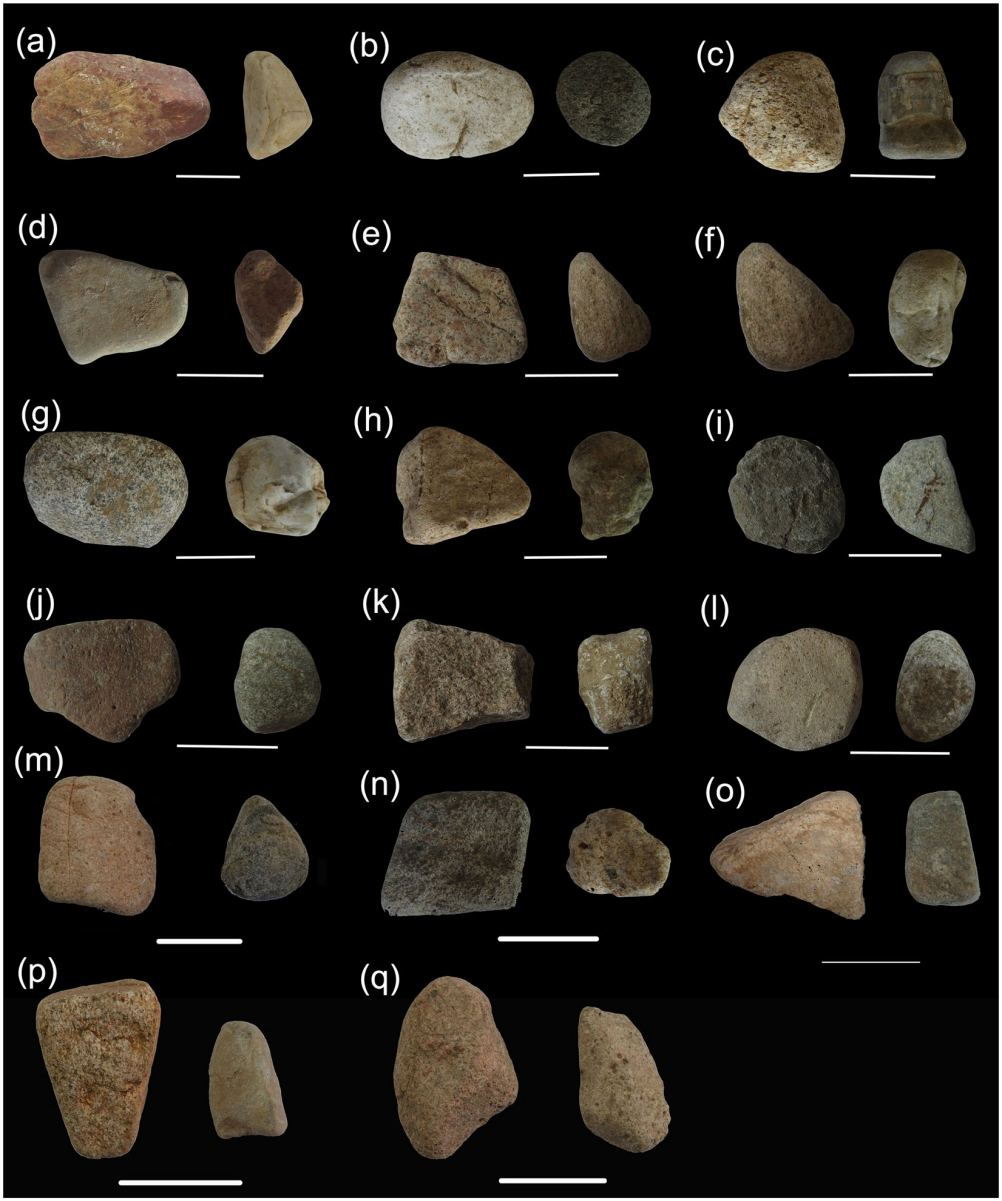
Modern stone tools used for dehusking and grinding foxtail millet, broomcorn millet and green bristlegrass. Scale bar: 5 cm. (**a**) Stones used for dehusking and grinding Qimengkelake (Xinjiang province). (**b**) Stones used for dehusking and grinding Gouzhuagu (Hebei province). (**c**) Stones used for dehusking and grinding Maomaodouzhimaliang (Shanxi province). (**d**) Stones used for dehusking and grinding Kaoshanmen (Jiangsu province). (**e**) Stones used for dehusking and grinding Zhushusu (Heilongjiang province). (**f**) Stones used for dehusking and grinding Mingu (Fujian province). (**g**) Stones used for dehusking and grinding Yugu (Henan province). (**h**) Stones used for dehusking and grinding Qutangxiaomi (Guangxi province). (**i**) Stones used for dehusking and grinding Huangkegouweisu (Hainan province). (**j**) Stones used for dehusking and grinding Zheng No. 468 (Henan province). (**k**) Stones used for dehusking and grinding Shaonong No. 11 (Inner Mongolia). (**l**) Stones used for dehusking and grinding Baigu (Tibet Autonomous Region). (**m**) Stones used for dehusking and grinding Jishu No. 1 (Hebei province). (**n**) Stones used for dehusking and grinding JinshuNo. 4 (Shanxi province). (**o**) Stones used for dehusking and grinding Jinsegouweicao (Beijing city). (**p**) Stones used for dehusking and grinding Judagouweicao (Hebei province). (**q**) Stones used for dehusking and grinding Zhouyegouweicao (Zhejiang province). All the images of stone tools were taken by Z. M..

In northern China, materials selected by ancient humans for making ground stone tools were typically easily collected and processed aggregated rock available in the immediate area. These resources mainly include malmstone, slate, and shale^2^. Resources available to us that best mimicked the material characteristics of the grinding stone tools excavated from the sites in northern China were sandstone and shale. We selected 17 sets of grinding stones and slabs from the area surrounding the farmland in the village of Xindian, Machikou town, Changping district of Beijing (40°09′N, 116°11′E; Fig. 8 and Table 2).

## Methods

### Methods for dehusking and grinding millets

#### Methods for dehusking millets

The seventeen sets of stones were cleaned with ultrapure water and a nylon brush to remove any dust that had adhered during storage. The stones were submerged completely in the ultrasonic bath and boiled in an ultrapure water bath for three hours. Next, the stones were processed separately in an ultrasonic bath after which they were baked in an electric drying oven at 100 degrees Celsius for three hours. Finally, the cylindrical stone of each group was used to husk the shells of millet caryopses, which were put on the flat sandstone, from side to side gently to let the seeds slip out from the bran and avoid damaging the millet seed coat (~52 min/millet lot).

#### Methods for grinding millets

The same seventeen sets of stone tools were cleaned in the manner described above. The stone from each group was used to break the millet seeds, which were put on the flat stone, by rolling the stone back and forth repeatedly, until the dehusked millet grain was reduced to flour (~17 min/millet lot).

### Starch grain extraction

#### Starch grains from millet seeds

Ten mature millet seeds selected from each sample of the seventeen millets were dehusked using clean tweezers and moved immediately into new, sterile test tubes in which they were soaked with ultrapure water for 12 h. The soaked seeds were then gently crushed with clean, glass, stirring rods to release the starches. The starch/water suspensions were then pipetted on to a clean glass slide, mounted in 10% glycerine and 90% ultrapure water, and then the cover glass was sealed with nail polish. For detailed protocol for extraction of starch grains from native millet seeds, please refer to ref.^23^.

#### Starch grains from residues on the surfaces of stones

The used and unused facets of each stone were cleaned with ultrapure water and sonicated for 10 min at a power of 40 kHz/200 W. A solution of 6% H_2_O_2_ was used for the oxidative breakdown of some of the larger particles. Next, the starch grains were isolated using a heavy liquid flotation with CsCl (density, 1.8 g/cm^3^). The recovered residue was mounted on a slide in 10% glycerine and 90% ultrapure water, then sealed with nail polish.

### Methods for Statistics and identification

#### Statistics

Previous studies have demonstrated that starch grains less than 5 μm long are difficult to detect via compound light microscopy at 400×, are rarely diagnostic for taxa, and occur in many plant tissues^27–31^. Therefore, observations and statistical analyses were only performed on starch grains that measured >5 μm. Because domesticated foxtail millet is the only plant in our previous experiments that has starch grains measuring >14.0 μm^23^, we also made note of the millet starches that measured >14.0 μm in this experiment. All slides prepared from all samples in this simulation experiment were examined using compound light microscopy and transmitted light microscopy. If the number of starch grains (>5 μm) in the slide was less than 100, we recorded, measured and counted every starch grain. If the number of starch grains (>5 μm) in the slide was more than 100, we examined the samples in the microscope first, and then found a clear view with unobscured and non-overlapping starch grains. Next we recorded, measured and counted every starch grain in the clear view. The numbers are somewhat variable due to the nature of the slides and the large amounts of starchy residues. To try to correct for this issue, if the number of starch grains in a field of vision under microscope did not reach 100, we then moved to the next field of view and counted until more than 100 starch grains were analyzed.

SPASS statistical software (Version 20.0) was used to analyze the starch data from both the grinding and seed extractions (Figs 2 and 5, Table 3).

#### Identification

Our starch classifications for modern millet starches emphasize attributes demonstrated by previous studies to be useful in identification^23^: shape, surface features, position and form of the hilum and fissures, number and characteristics of pressure facets, presence or absence of demonstrable lamellae and mean maximum length averaged from the measurement of more than 100 grains. The main variables recorded include changes in shape and size of grains, surface modifications, visibility of lamellae and change in the extinction cross. Details of the millet starch grain classification are presented in our previous study in ref.^23^.
